# Supplementary material for: A proteomic network approach across the ALS‐FTD disease spectrum resolves clinical phenotypes and genetic vulnerability in human brain
Source: EMBO Mol Med. 2017 Nov 30;10(1):48–62. doi: 10.15252/emmm.201708202 (PMC5760858; doi:10.15252/emmm.201708202)
Supplement: Supplementary file 1 — Expanded View Figures PDF [file EMMM-10-48-s001.pdf]

Expanded View Figures

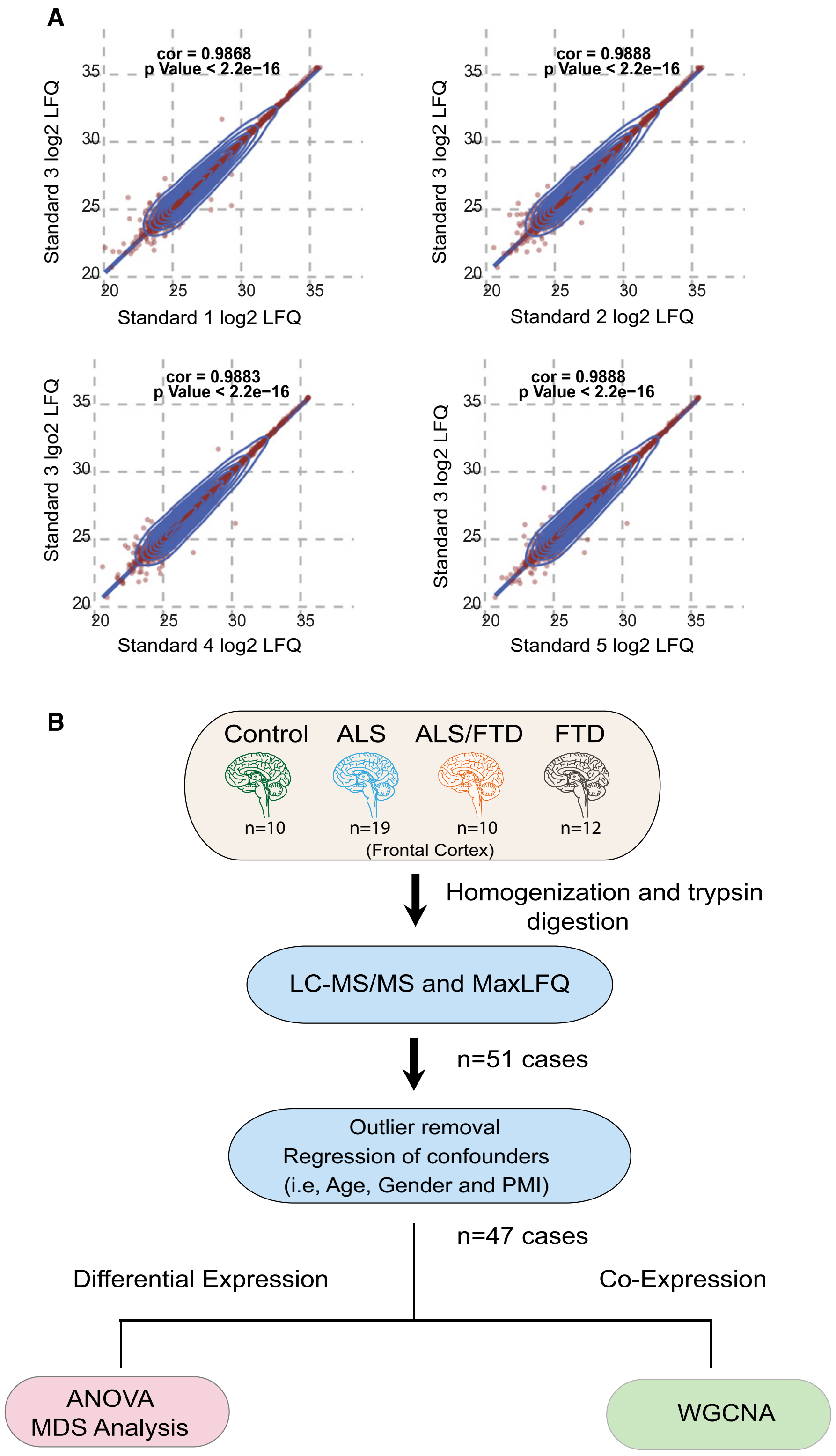

Figure EV1. Proteomic quality control measures and data analysis pipeline.

A Pooled homogenates of several neurodegenerative disease brains were used as a technical standard and analyzed at several points within the MS/MS batch. Pearson correlation plots of LFQ data compared across each pair of pooled standard LC-MS/MS runs (n = 2,299 overlapping proteins) were generated with calculated rho (cor) and P-values as shown.

B Workflow for data collection, pre-processing, and analysis prior to differential and co-expression analysis.

© 2017 The Authors

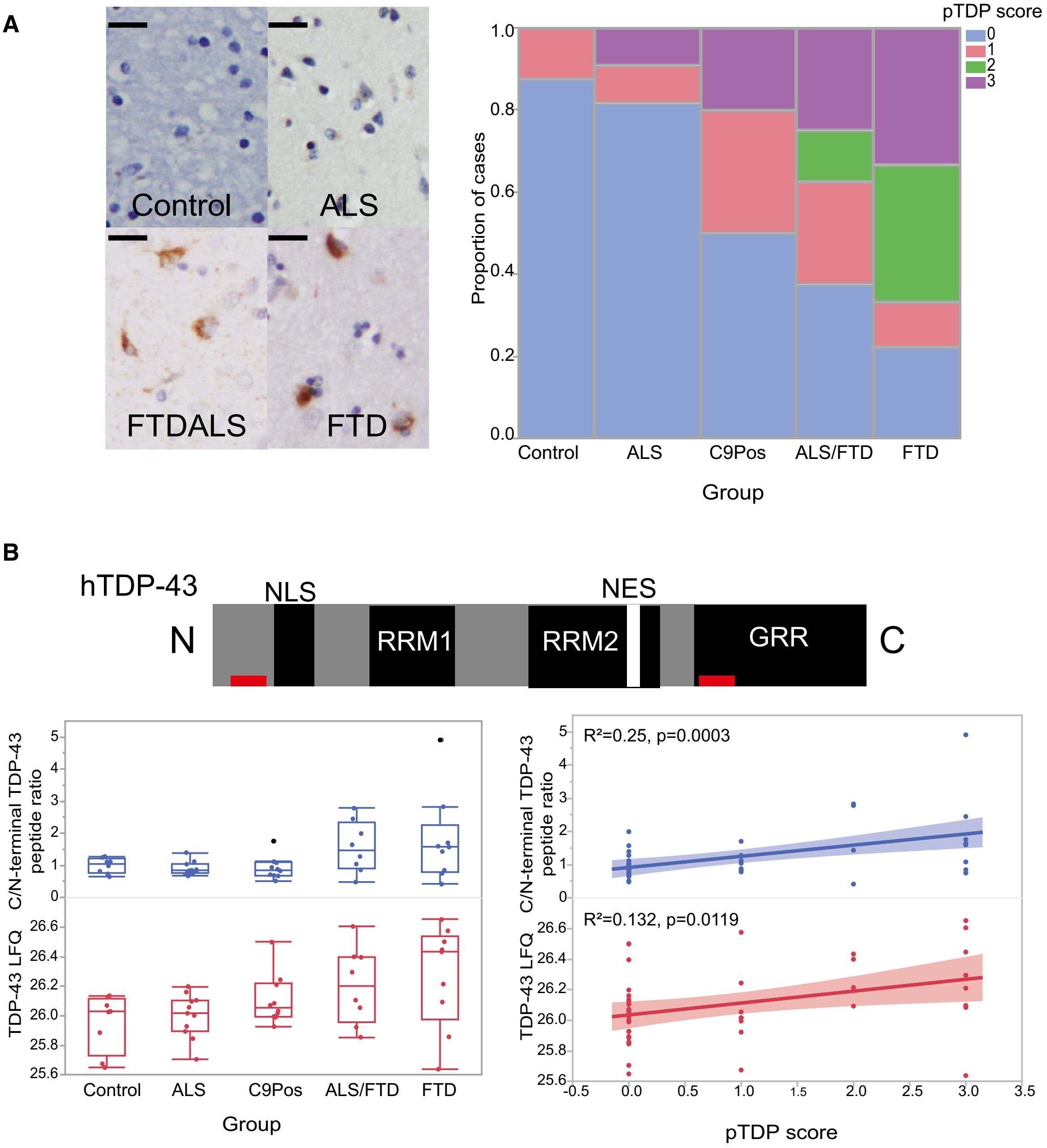

Figure EV2. TDP-43 pathological and proteomic profiles across clinical groups.

A Representative images display phosphorylated TDP-43 (pTDP) pathology in the frontal cortex of patient brain samples (Scale bar = 20 μm). All cases included in the analysis were given a pTDP pathological rating score (pTDP score), which is summarized in the mosaic plot to the right.

B The human TDP-43 domain structure is shown, including its nuclear localization signal (NLS), RNA recognition motifs, nuclear export signal (NES), and glycine-rich regions (GRR). The C-terminal (FGGNPGGFGNQGGFGNSR; residues 276–293 of human TDP-43, UniProtKB Q13148-1) and N-terminal (LVEGILHAPDAGWGNLVYVVNYPK; residues 56–79 of human TDP-43, UniProtKB Q13148-1) peptides used to generate a C/N-terminal peptide ratio are indicated by red rectangles. Box plots show graphical representations of the C/N-terminal peptide ratio across the groups (blue). Also, shown below are box plots representing total TDP-43 (TDP-LFQ) levels across the groups (red). A one-way ANOVA was conducted to compare this ratio across the groups (control (CTL), ALS, FTD/ALS, and FTD). One-way ANOVA comparing the C-terminal peptide extracted ion chromatogram (XIC), representing abundance, to N-terminal peptide XIC ratio at the $P \leq 0.05$ level [$F(3, 43) = 2.6508$, $P = 0.0607$] showed a trend toward increased ratio in FTD cases. There was a significant difference by one-way ANOVA comparing TDP-LFQ levels across the clinical groups at the $P \leq 0.05$ level [$F(3, 43) = 3.0530$, $P = 0.0385$]. Correlation plots are shown to the right which display correlation between the C-terminal peptide XIC to N-terminal peptide XIC ratio (blue), TDP-LFQ (red), and pTDP pathological rating; both traits were significantly correlated to pTDP score ($R^2 = 0.251$, $P = 0.0003$ and $R^2 = 0.132$, $P = 0.0119$, respectively). Shaded areas in the correlation plot represent 95% confidence intervals. Box plots depict mean (horizontal bars) and variance (25th to 75th percentiles), with whiskers extending to the last non-outlier measurements, as shown.

© 2017 The Authors

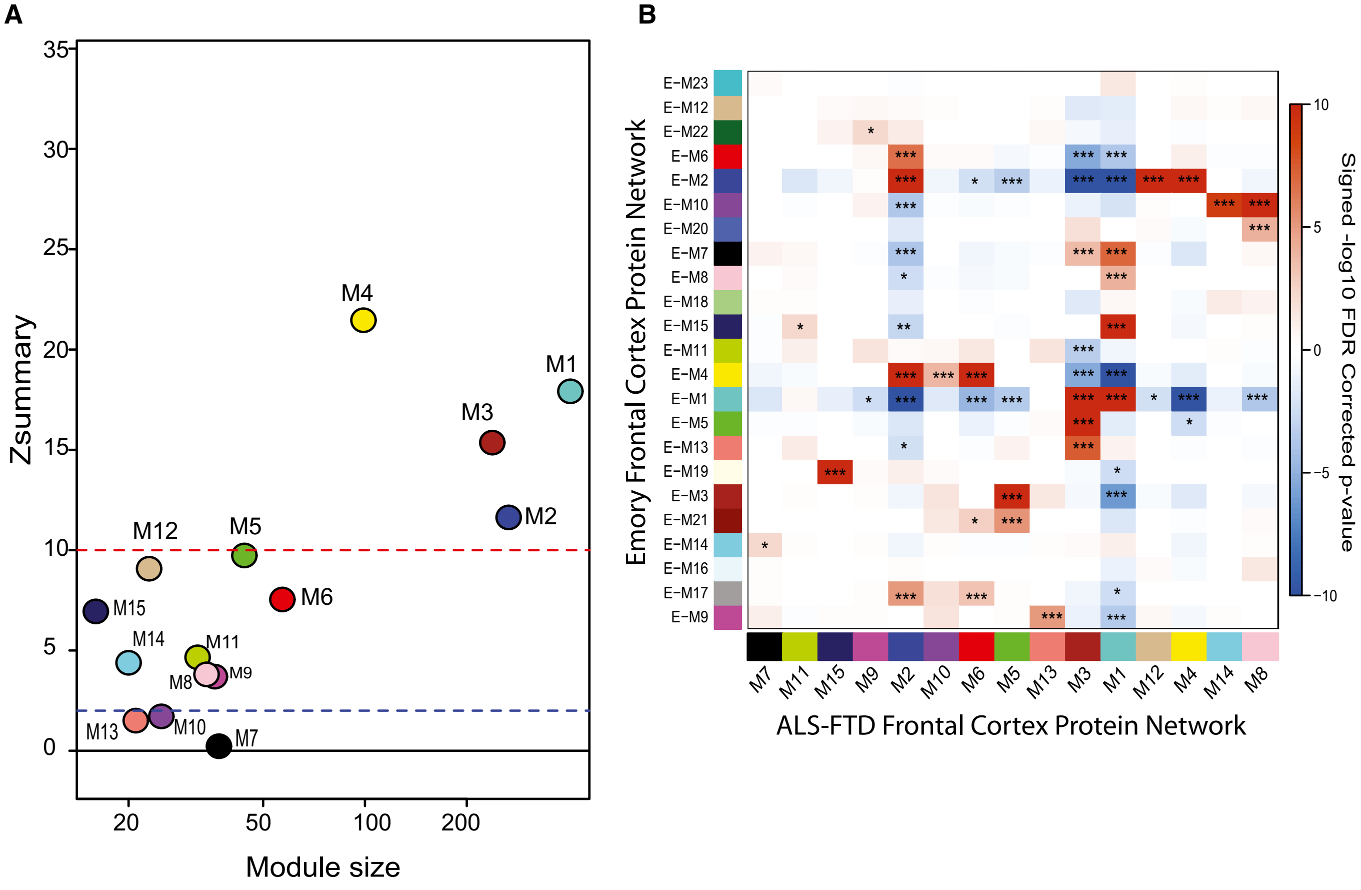

Figure EV3. Assessing module preservation of the ALS-FTD protein network.

A Preservation Z-summary test of protein modules in frontal cortex comparing total homogenate proteome to previously generated Emory Brain Network from label-free proteomic analysis of the frontal cortex from controls, Alzheimer's disease (AD), Parkinson's disease (PD), and ALS cases (Seyfried *et al*, 2017). Less than 0 represents no preservation; 0–2, weak preservation; 2–10, moderate preservation; and more than 10, high preservation. 12 of the 15 modules generated in the total homogenate proteomic network were preserved in the Emory Proteomic network, eight had moderate preservation with a Z-summary score between 2 ($P < 0.05$) (blue dotted line) and 10 ($P < 0.01$) (red dotted line), while four were highly preserved with a Z-summary score greater than 10 ($P < 0.01$).

B Over-representation analysis for protein networks generated from frontal cortex tissue (Emory Brain Proteomes) using a two-sided Fisher exact test with 95% confidence intervals. *P*-values were FDR adjusted to account for multiple comparisons. The 15 modules in the total homogenate network were aligned to the 23 modules from the previous Emory brain network. Colors represent whether module gene symbol lists showed significant overlap (red), depletion (blue), or no significant under-/over-representation (white) for protein membership. Numbers displayed on the heatmaps represent positive signed −log10(FDR-adjusted *P*-values), and asterisks represent the level of significance of comparisons (**P* < 0.05; ***P* < 0.01, ****P* < 0.005).

Figure EV4. Validation of astrocyte and microglial proteomic markers that increase across the ALS-FTD spectrum.

A Western blot analysis for the astrocytic, HEPACAM and GFAP, and microglia, MSN and TTP1, hub proteins (M6) across randomly selected cases from control ($n = 5$), ALS ($n = 12$), ALS/FTD ($n = 6$) and FTD ($n = 7$) groupings. Both HEPACAM and TPP1 display high molecular weight species (indicated by asterisk *), representing well-established glycosylation events on these proteins, respectively (Golabek *et al*, 2003; Moh *et al*, 2005), which were included in the quantification. Western blot for GAPDH is provided as a loading control (bottom panel). Microglial and astrocyte markers are highlighted in blue and green, respectively.

B Quantification was performed by ImageJ and statistical significance determined by ANOVA with Dunnett's *post hoc* test to compare each disease group to the control group (**P*-value < 0.05). Box plots depict mean (horizontal bars) and variance (25th to 75th percentiles), with whiskers extending to the last non-outlier measurements, as shown.

C Representative immunohistochemistry images of cortex and white matter regions of the frontal cortex from case 20 (ALS) and case 30 (ALS/FTD). GFAP and HEPACAM staining in cells of the cortical layers demonstrate staining intensity in astrocytes in the frontal cortex, whereas MSN and TPP1 staining show a microglial expression pattern. The relative increase in immunoreactivity for GFAP, HEPACAM, MSN, and TPP1 in the ALS/FTD case likely reflects changes in the abundance, of astrocytes and microglia measured by proteomics in module 6 (red). Scale bars represent 50 μm in low-power images and 20 μm in insets.

 © 2017 The Authors

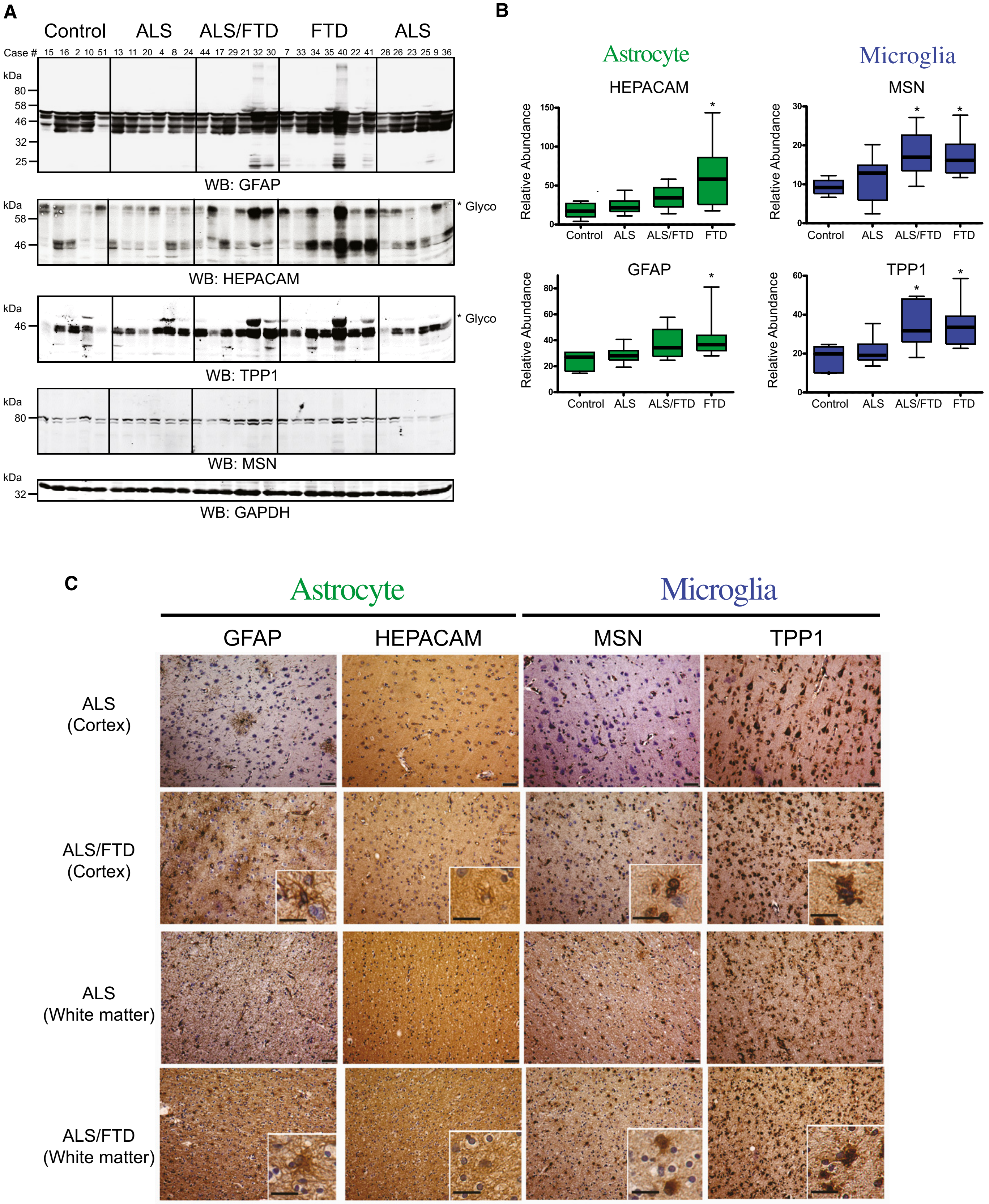
A
Control
ALS
ALS/FTD
FTD
ALS
Case #
kDa
WB: GFAP
* Glyco
WB: HEPACAM
WB: TPP1
WB: MSN
WB: GAPDH
B
Astrocyte
Microglia
HEPACAM
MSN
GFAP
TPP1
Relative Abundance
Control
ALS
ALS/FTD
FTD
C
Astrocyte
Microglia
GFAP
HEPACAM
MSN
TPP1
ALS (Cortex)
ALS/FTD (Cortex)
ALS (White matter)
ALS/FTD (White matter)

Figure EV4.

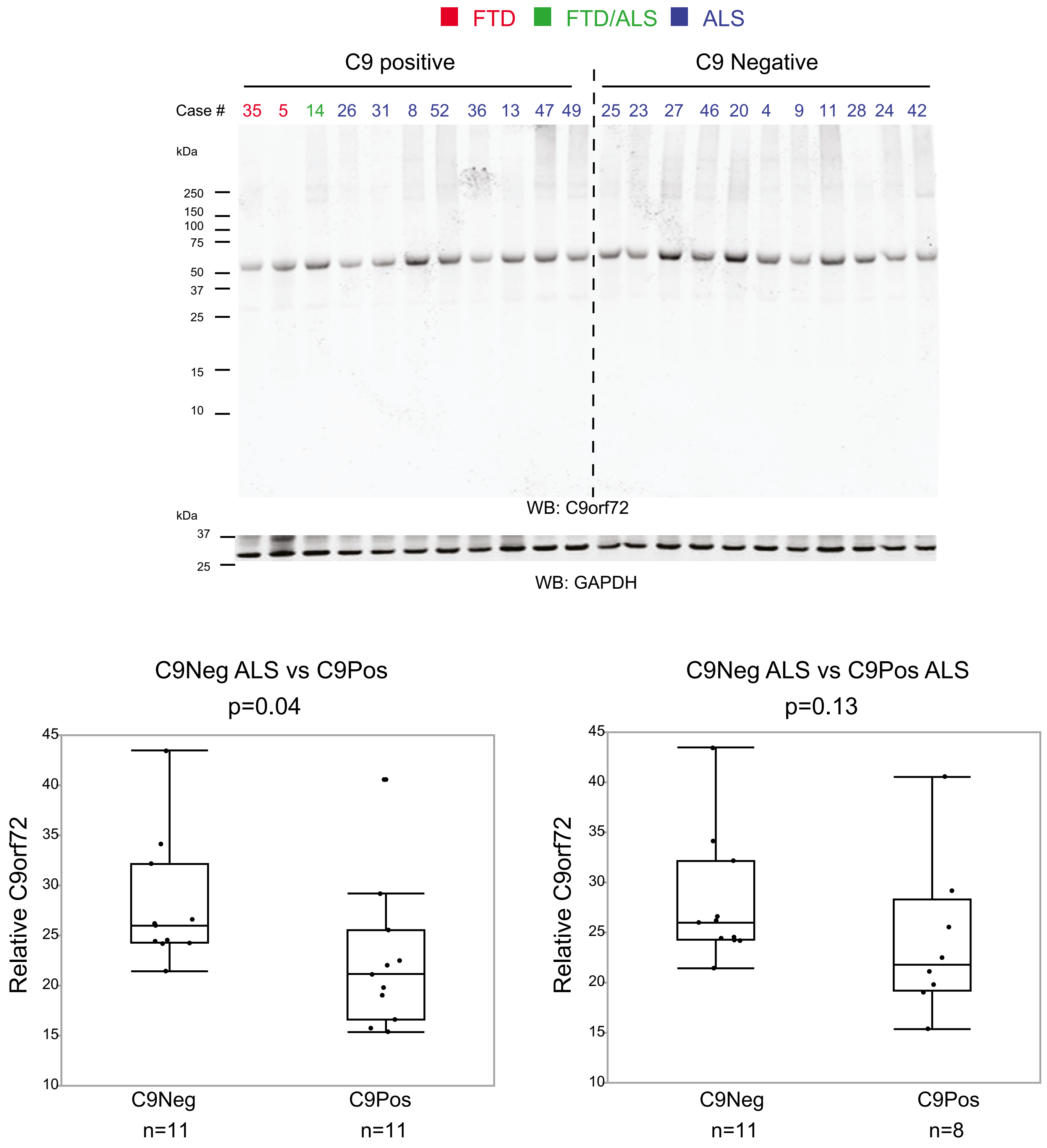

Figure EV5. C9orf72 protein levels are reduced in C9Pos (+) compared to C9Neg (−) ALS cases.

Western blot analysis probing C9orf72 protein (~55 kDa) across individual C9Pos and C9Neg ALS cases. A decrease in C9orf72 protein expression in C9pos cases demonstrated by quantitative difference in protein expression, relative to GAPDH protein loading control (*t*-test *P*-value = 0.04 comparing 11 C9Neg ALS cases to 11 C9Pos cases, *t*-test *P*-value = 0.13 comparing 11 C9Neg ALS cases to 8 C9Pos ALS cases). Box plots depict mean (horizontal bars) and variance (25th to 75th percentiles), with whiskers extending to the last non-outlier measurements, as shown.

© 2017 The Authors
